# Catheter-Based Therapies and Other Management Strategies for Deep Vein Thrombosis and Post-Thrombotic Syndrome

**DOI:** 10.3390/jcm9051439

**Published:** 2020-05-12

**Authors:** Siddhant Thukral, Suresh Vedantham

**Affiliations:** 1 School of Medicine, University of Missouri—Kansas City, Kansas City, MO 64108, USA; stry9@mail.umkc.com; 2 Mallinckrodt Institute of Radiology, Washington University in St. Louis, St. Louis, MO 63110, USA

**Keywords:** post-thrombotic syndrome, deep vein thrombosis, catheter-directed therapies, superior vena cava syndrome, endovascular therapy, axillosubclavian DVT

## Abstract

Acute deep vein thrombosis (DVT) causes substantial short-term and long-term patient morbidity. Medical, lifestyle, and compressive therapies have been investigated for the prevention of pulmonary embolism (PE) and recurrence of venous thromboembolism (VTE). However, patient-centered outcomes such as resolution of presenting DVT symptoms and late occurrence of post-thrombotic syndrome (PTS) have not been prioritized to the same degree. Imaging-guided, catheter-based endovascular therapy has been used in selected patients to alleviate these sequelae, but important questions remain about their optimal use. In this article, we review the available evidence and summarize the rationale for use of catheter-based therapy in specific patient groups.

## 1. Introduction

An acute deep vein thrombosis (DVT) refers to an episode of venous thrombosis for which symptoms have been present for 14 days or less or for which imaging studies confirm that the venous thrombosis occurred within the last 14 days [1]. With an estimated population incidence of 1:1000 persons each year, acute DVT of the lower extremities is associated with significant morbidity and a high rate of recurrence [2,3,4,5]. Frequently presenting with non-specific symptoms such as pain, swelling, and tenderness of the affected extremity, the early course of DVT can vary among patients, ranging from (1) complete resolution, (2) development of severe complications such as acute limb threat, renal vein thrombosis, or Budd–Chiari syndrome (BCS), or even (3) death by pulmonary embolism (PE) [6,7,8,9,10]. Anticoagulant therapy is usually effective in preventing these complications; however, even with optimal initial management, there remains a substantial risk of developing post-thrombotic syndrome (PTS), including or not including venous ulcers, as a long-term complication [11,12,13].

The broad consensus around the need for excellent acute care has contributed to a common misunderstanding of DVT as an ephemeral condition that consists of one or several discrete clotting events [14]. Current DVT care guidelines define a standard of care that is highly effective at preventing PE and venous thromboembolism (VTE) recurrence but often neglect to keep the holistic picture of patient-centered care in mind [15,16]. Specifically, less consideration tends to be given to a patient’s experience with the symptoms of DVT, early recovery of function, and the individualized potential to develop PTS, a common, chronic complication that can substantially impair patients’ quality of life (QOL) [11,12,13].

Catheter-based therapies for DVT were introduced in the 1990s and have been used selectively, with the primary intent of addressing these patient-centered objectives [17]. The purpose of this article is to provide guidance to practitioners on optimal patient selection for these more aggressive approaches, in a manner that is informed by the results of recent randomized trials.

## 2. Categorizing Patients with Acute DVT

Treatment for DVT will vary based on clot extent, thrombosis history, and comorbidities. Because the risk of developing serious complications such as PE is highest near the time of diagnosis, guidelines recommend the initiation of anticoagulation therapy in patients with high suspicion of DVT even before ultrasound confirmation of diagnosis, in the absence of major risk factors for bleeding [16,18].

Due to differences in the risk of developing PE, distal DVT (involving deep veins below the popliteal vein) is managed differently from proximal DVT (involving the popliteal and/or more cephalad deep veins) [19]. As noted in previous research, patients with proximal DVT generally require therapeutic-dose anticoagulation for at least 3 months [15]. However, less well-appreciated is another anatomic distinction of importance: the subset of proximal DVT patients who have thrombus involvement of the iliac and/or common femoral vein (“iliofemoral DVT”) actually have the highest rates of VTE recurrence, PTS, and more severe PTS [19,20,21,22,23]. Since the vast majority of cases of iliac vein thrombosis extend into the common femoral vein, most of these patients are easily identified on the initial diagnostic ultrasound exam. Hence, it is the authors’ view that patients with lower-extremity DVT can and should be grouped into three anatomic categories based on their future risk of PE and PTS: distal DVT, proximal (femoral–popliteal) DVT, or proximal (iliofemoral) DVT.

Clinical severity is also an important consideration. Patients with asymptomatic (incidentally discovered) DVT are at very low risk of developing PTS [24]. In contrast, patients with more severe presenting symptoms have greater impairment of early QOL; patients who are highly symptomatic 1 month after diagnosis are at high risk for PTS [23,25]. Finally, patients who present with tense limb swelling with cyanosis should undergo urgent vascular evaluation, since patients with phlegmasia cerulea dolens (while rare) are at high risk for limb amputation.

## 3. Impact of Anticoagulation on PTS Occurrence

Although a detailed exposition of anticoagulant options is beyond the scope of this review, it is important to understand the advantages and limitations of anticoagulant therapy in terms of patient-centered outcomes. First, contemporary prospective studies show that despite the use of anticoagulation, one-third of patients do not recover their baseline QOL after their DVT [23]. Second, the risk of developing of PTS is increased by the recurrence of ipsilateral DVT [26,27]. Third, insufficient anticoagulation during the first months following diagnosis is correlated with an increased risk in recurrent ipsilateral DVT and PTS [28,29,30]. Therefore, it is reasonable to conclude that beyond prevention of PE, quality anticoagulant therapy is also important for the reduction of PTS.

Nevertheless, nearly 50% of patients with proximal DVT develop PTS over 2 years [20,31,32]. While direct oral anticoagulants (DOACs) do not substantially decrease the risk of symptomatic recurrent VTE compared to warfarin, their ease of use is speculated to increase compliance and thereby reduce recurrent (subclinical) thrombus formation and PTS [15]. However, studies comparing DOACs to warfarin for PTS prevention have had methodological limitations and inconsistent results, with the more rigorous studies failing to find significant differences [33,34,35,36].

Outpatient therapy for acute DVT is safe and effective for the majority of patients [6,37], but can reduce patient–physician interaction time and contribute to: (1) the progress of a patient’s recovery from presenting DVT symptoms being overlooked; (2) a failure to ensure that high-risk patients (e.g., those with iliofemoral DVT) are truly adhering to therapy; and (3) inadequate patient education on long-term DVT morbidity, risk factor modification measures, and potential treatment options [38].

## 4. Compression Therapy

Elastic compression stockings (ECSs) increase venous return through external compression, reducing edema and increasing the efficiency of the calf muscle pump [35]. Recently, the utility of compression therapy for PTS prevention has been called into question. In the multicenter, randomized, double-blind, placebo-controlled Compression Stockings to Prevent the Post-Thrombotic Syndrome (SOX) trial (*n* = 803), no difference was observed in the occurrence of PTS between the active elastic compression stocking (ECS) group and the placebo ECS group through 750 days (Hazard Ratio (HR): 1.13; 95% Confidence Interval (CI) 0.73–1.76; *p* = 0.58). Additionally, increased length of treatment with ECS does not appear to confer advantages as two multicenter, single-blind, randomized controlled trials failed to show superiority of 24 month ECS therapy compared with shortened therapy for the primary outcome of PTS incidence [39,40].

Nevertheless, ECSs are helpful in reducing leg swelling in some patients. Adoption of proactive strategies can increase compliance. Due to increased leg tenderness and larger fluctuations in edema shortly after DVT, compression wraps may be more effective and better tolerated initially than sized-to-fit ECS. After 7–14 days, the leg may be fitted for ECS. Starting at lower ankle pressure (20–30 mmHg) knee-high ECS and moving up incrementally to higher pressure and length may be an effective strategy in increasing compliance. Patients should be questioned at each visit about barriers to achieving compliance with compression therapy such as discomfort with use, cosmetic concerns, or difficulty remembering to use them. Such problems should be addressed on an individual basis. Given that the best available evidence does not support a PTS prevention effect, the prescription of stockings should not be dogmatically instituted, but should be tailored toward reducing symptoms and enhancing function as dictated by the individual patient’s needs and experiences [31].

## 5. Ambulation and Lifestyle Intervention

For many years, it was believed that ambulation following acute DVT increases the risk of thrombus dislodgment and PE. However, meta-analysis of contemporary studies suggests that this is not the case [41,42]. Additionally, one small (*n* = 53) randomized trial of patients with acute proximal DVT suggested that early ambulation may reduce the incidence and severity of PTS [43,44]. Overall, once anticoagulation treatment has been initiated, early ambulation, as tolerated, may be encouraged.

## 6. Endovascular Therapy for the Management of Acute DVT

Despite the use of anticoagulant therapy and ECS, PTS incidence in patients with proximal DVT is estimated to range between 25% and 50% at 2 years [23,32]. Many patients suffer impaired health-related QOL, and the development and severity of PTS have been shown to represent leading contributors to poor QOL in DVT patients [41,42,45,46,47].

The “open vein hypothesis” refers to the idea that early active removal of venous thrombus may enable preservation of valvular function and venous patency and prevention or reduction of PTS [5,20,48,49]. The importance of early thrombus resolution is supported by clinical studies such as the prospective cohort study of 313 patients by Prandoni et al. [50], in which patients with residual thrombus on 6 month follow-up ultrasound were found to be at significantly increased risk of recurrent VTE (HR 2.4; 95% CI: 1.3–4.4; *p* = 0.004). Similarly, in an observational study of 316 initially presenting DVT patients by Young et al. [51], patients with residual thrombus after completion of oral anticoagulant therapy were observed have a higher risk of thromboembolic recurrence compared to patients with completely clear vessels (HR: 2.2; 95% CI: 1.19–4.21; *p* = 0.012). In a systematic review of 11 randomized anticoagulation trials, Hull et al. [52] reported a strong correlation (correlation = 0.81, *p* = 0.005) between quantitative thrombus burden change during initial DVT treatment, determined through imaging assessments, and recurrent VTE. Moreover, a subgroup analysis from a single-center randomized trial evaluating the use of compression stockings in patients with proximal DVT, found that the presence of residual thrombus or valvular reflux on 6 month follow-up ultrasound was associated with an increased risk of developing PTS [53]. Randomized trials of systemic thrombolysis and surgical venous thrombectomy have also suggested that rapid thrombus clearance is associated with improved long-term symptom outcomes; however, these therapies were associated with substantial risks and the studies had substantial methodological limitations [49,54,55].

The use of percutaneous endovascular methods to eliminate the thrombus and restore venous patency (collectively termed “endovascular DVT thrombolysis” in this review) was introduced by interventional radiologists nearly 30 years ago [17,56]. The basic premise is that by targeting therapy directly into the thrombus using imaging guidance, therapy may be more effective and safer (due to the reduced dose of fibrinolytic drugs). Gradual evolution in technique, safety-oriented elements, and device innovation have increased the precision by which these modalities can be utilized in recent years [14]. There is a plethora of specific therapies that can be applied, but they can be broadly grouped as follows: (1) catheter-directed thrombolysis (CDT) refers to the direct intra-thrombus delivery of a fibrinolytic drug using a catheter or device that is positioned within the thrombosed vein using imaging guidance. Drugs that have been used for this purpose include recombinant tissue plasminogen activator (rt-PA), urokinase, streptokinase, reteplase, and tenecteplase; (2) percutaneous mechanical thrombectomy (PMT) refers to the utilization of catheter-mounted devices for the mechanical aspiration or maceration of the thrombus; and (3) pharmacomechanical catheter-directed thrombolysis (PCDT) refers to the combined use of CDT and PMT in any of several forms [1].

Studies have demonstrated that each of the above methods is able to substantially reduce thrombus burden in patients with acute DVT. However, before any patient can be considered for endovascular DVT thrombolysis, measures to ensure optimal patient selection and during-procedure patient safety are essential. Most importantly, because fibrinolytic drugs can cause serious bleeding, a careful review of the patient’s medical history and current condition must be conducted to identify factors that may increase the risk of bleeding. This may include active bleeding; recent (<7–10 days) major trauma, surgery, obstetrical delivery, or other invasive procedure; severe thrombocytopenia; advanced age (bleeding risk increases after 65 years of age); or the presence of lesions in critical locations that can bleed (e.g., brain/spine metastases from cancer). Patients with cancers known to metastasize to the central nervous system should be considered for brain imaging prior to undertaking thrombolytic therapy. Because a majority of indications for DVT thrombolysis are elective, most patients at higher bleeding risk should not undergo thrombolytic therapy.

For patients who do undergo endovascular DVT thrombolysis, relevant safety precautions can include the following: (1) administering sufficient preprocedure hydration to patients with pre-existing renal insufficiency; (2) administering steroids and antihistamine agents premedication to patients with contrast medium allergies; (3) routinely monitoring vital signs and oxygen saturation during the procedure; (4) using sterile technique; and (5) routinely using ultrasound guidance to obtain venous access [57]. The physician operator should know the status of the patient’s anticoagulant therapy and should adjust the start time of the procedure with careful consideration of when the drug was last given, the partial thromboplastin time (PTT) for heparin recipients, and the international normalized ratio (INR) for warfarin recipients, if applicable.

The management of DVT in patients with severe thrombocytopenia is complex and lacks guidance from large prospective studies. As per Society of Interventional Radiology (SIR) quality improvement guidelines [57], patients with severe thrombocytopenia (there is no established cutoff but we suggest <75,000/cm^3^ for most patients) should not receive thrombolytic therapy beyond exceptional circumstances; if this is necessary, then the dose of fibrinolytic drug should be minimized, and concomitant anticoagulation should be maintained at a very low level or avoided entirely. For patients with heparin-induced thrombocytopenia who subsequently develop DVT, argatroban or bivalirudin may be acceptable alternatives to heparin during treatment with CDT [58,59]. For other patient groups, direct data or guidance are lacking.

In recent years, the utility of endovascular DVT thrombolysis to improve clinical outcomes has been investigated through a series of randomized controlled trials. It is important to note that these trials have only addressed the first-line use of endovascular thrombolysis for initially presenting DVT; however, aggressive therapy is often contemplated in a number of additional real-world scenarios, as noted below.

### 6.1. Acute Limb Threat

Phlegmasia cerulea dolens (PCD) is diagnosed when there is near-complete obstruction of venous flow from the limb leading to impaired arterial inflow, which can lead to limb-threatening compartment syndrome [60]. Most PCD patients have a large iliofemoral DVT, and some have additional involvement of the inferior vena cava (IVC) [61]. PCD is usually associated with tense swelling of the limb, pain out of proportion, and cyanosis [60,62]. In small case series and case reports, PCD has frequently led to death (40%), limb amputation (50%), and PE (22%) despite the use of anticoagulant therapy [60]. For this reason, treatment escalation is common—many patients undergo emergent fasciotomy (to rapidly reduce limb compartment pressures) and either surgical thrombectomy or endovascular DVT thrombolysis (to rapidly debulk the thrombus and restore venous outflow) [63,64]. Because these patients can be extremely ill, urgent endovascular therapy is usually reasonable as long as there are no major risk factors that would increase the bleeding risk with thrombolytic therapy. Even when aggressive therapy is instituted rapidly, there remains some risk of limb loss and death due to progressive ischemia and reperfusion injuries [65].

### 6.2. Visceral Organ Risk

Acute renal vein thrombosis (RVT) is a rare but well-defined complication of nephrotic syndrome, renal transplant, and large iliocaval DVT. Patients with acute RVT can present with symptoms such as acute flank pain, gross hematuria, and worsening renal function and have high risk of allograft loss in case of kidney transplant [8,66]. Although anticoagulation is the first-line therapy for most cases of RVT, and although many patients will develop reasonable collateral flow even if the renal vein outflow remains obstructed, small case series attest to the ability of endovascular DVT thrombolysis to rapidly restore renal vein flow [8,66,67,68]. This option may be appropriate to consider when a few days of anticoagulation has failed to produce clinical improvement, especially when there is a solitary functioning kidney, since it cannot be known in advance whether the degree of future collateralization will support adequate renal function.

Budd–Chiari syndrome (BCS) represents a heterogenous group of disorders that result in venous obstruction of the hepatic vein and/or retrohepatic IVC [9,69]. For patients with BCS, priority is given to the rapid removal of the hepatic vein obstruction to reduce portal venous pressure and progression of cirrhosis, hepatic dysfunction, and esophageal varices [9]. Case series have identified benefits from a minimally invasive approach [69,70,71]. For example, one retrospective study (*n* = 108) reported a technical success rate of endovascular treatment of greater than 99% and a cumulative 10 year primary patency of 79%, with infrequent procedure-related complications [69]. In some clinical scenarios, trans-jugular portosystemic shunt placement is also part of the approach.

### 6.3. Axillosubclavian DVT and Superior Vena Cava Syndrome

Superior vena cava syndrome (SVCS) results from the mechanical obstruction of venous blood flow through the superior vena cava (SVC) [72]. SVCS is classically associated with symptoms such as edema of the face, neck, upper extremities; shortness of breath and cough; distended veins in head and neck; flushing of face and neck; and headache and hoarseness [72]. These symptoms are typically worse in a supine position with variable degree of relief when the patient sits upright [73]. Though a majority of SVCS cases are secondary to a malignant process, benign causes such as mediastinal fibrosis, pacemaker lead implantation, and central venous catheter insertion are increasingly prevalent and estimated to represent up to 40% of cases [74,75]. Treatment options will vary based on the underlying pathology of SVCS but range from medical and radiation therapy to endovascular and surgical therapy [76].

SVCS can be a chronic condition or can result from acute thrombosis; in the latter situation, CDT followed by anticoagulation may be effective if used soon after acute symptom onset (e.g., within 7–10 days) [73,77]. While small studies have shown encouraging results with CDT, the added risk of bleeding must be considered alongside any potential benefit and patients should be followed closely after the procedure to ensure adequate symptom response [77,78,79].

Primary axillosubclavian vein DVT, also known as Paget–Schroetter syndrome or “effort thrombosis”, is caused by extrinsic venous compression at the anterior portion of the bony thoracic outlet and most typically affects younger patients who engage in athletic activities that involve repetitive motion of the shoulder girdle [80]. Patients may present with symptoms of chest and upper-extremity pain with engorgement of superficial veins on the affected areas [80]. Treatment of acute primary axillosubclavian DVT with anticoagulation alone is often ineffective leading to significant residual symptoms and chronic disability of the affected limb [81]. In modern practice, the preferred approach for such patients is early endovascular DVT thrombolysis (within 2 weeks of symptom onset) followed by surgical thoracic outlet decompression, assuming there are no contraindications [80,81].

Upper-extremity DVT can also be secondary to other causes including intravascular devices (e.g., central venous catheters, pacemakers), cancer, or other coagulopathies. Most such patients are adequately managed with anticoagulation alone. PTS does occur in the upper extremity, but less frequently than in the lower extremity, and is more likely to be significant in patients with axillosubclavian DVT in the dominant arm [30]. Because natural collateralization results in symptom improvement in most patients, endovascular DVT thrombolysis is reserved for patients whose symptoms progress despite anticoagulation and who are at low risk for bleeding.

### 6.4. Severe Symptoms despite Anticoagulation for Lower-Extremity DVT

Among patients diagnosed with DVT and anticoagulated, a minority of patients will have worsening or non-improving symptoms with severe functional limitation. Typically, these patients will have iliofemoral DVT. In the setting of unremitting or worsening symptoms, endovascular DVT thrombolysis is reasonable to consider for the purpose of providing early symptom improvement and ease of ambulation in patients who are a low risk for bleeding and who are motivated to experience more rapid symptom improvement and to accept the attendant slight increase in bleeding risk. This “secondary, selective” use of catheter-based therapy is indirectly supported by the findings of the Acute Venous Thrombosis: Thrombus Removal with Adjunctive Catheter-Directed Thrombolysis (ATTRACT) trial, which reported better early DVT symptom resolution and improved early QOL for iliofemoral DVT patients with use of PCDT compared with anticoagulation alone [82].

### 6.5. Initially Presenting DVT

Unlike the above situations in which endovascular DVT therapy is used very selectively, it has been proposed that a much larger number of patients who present with symptomatic proximal DVT should be managed with endovascular DVT thrombolysis in addition to their anticoagulant therapy, with the main goal of preventing or reducing PTS. However, whether or not the safety downsides (e.g., rare intracranial bleeds) could be justified in a much larger number of patients has been the topic of controversy. Fortunately, the conclusion of three recent, well-designed, multicenter randomized controlled trials has provided insight into the overall utility of this proposition.

In the Norwegian Catheter-Directed Venous Thrombolysis (CaVenT) trial, patients (*n* = 209) with acute proximal DVT who were treated with traditional infusion CDT, anticoagulation, and compression had a reduction in 2-year PTS (41.1% vs. 55.6%; *p* = 0.047) compared with anticoagulation and compression alone [83]. For patients randomized to CDT, the median time from symptom onset to CDT was 6.4 days [84]. The PTS reduction with CDT increased at the study’s 5-year follow-up, but no difference in health related QOL beyond 6 months was observed between the two treatment arms [84,85].

Subsequently, the ATTRACT study evaluated the addition of PCDT to compression and anticoagulation in patients with symptomatic proximal DVT involving the femoral, common femoral, and/or iliac vein. In this larger (*n* = 692) study, PCDT did not reduce the cumulative 2-year occurrence of PTS in the overall study population or in its iliofemoral (risk ratio, 0.95; 95% CI, 0.78–1.15; *p* = 0.59) or femoral–popliteal subgroups (risk ratio  =  0.97; 95% CI, 0.75–1.24; *p* = 0.79) [20,86,87]. The median duration of symptoms at randomization for the entire ATTRACT trial was 6 days, and PCDT-arm patients had the procedure performed at a median of 1 day post-randomization [20]. Subgroup analysis supported the conclusions that (a) PCDT provided no benefits in patients with DVT limited to the femoral and popliteal veins; (b) patients with iliofemoral DVT experienced reduced PTS severity, a reduced occurrence of moderate-or-severe PTS, and improved venous disease-specific health-related QOL out to 24 months compared with non-lysed patients, with the largest benefits seen within the first 6 months; and (c) PCDT is not cost effective as an initial treatment strategy for proximal DVT or femoral–popliteal DVT, but may represent intermediate-value care for patients with iliofemoral DVT, particularly if additional studies enable further understanding of which patients benefit the most from PCDT therapy [20,82,86,87,88].

In the Dutch Ultrasound-Accelerated Catheter-Directed Thrombolysis Versus Anticoagulation (CAVA) trial, which evaluated 184 patients with acute iliofemoral DVT, ultrasound-assisted CDT did not reduce the occurrence of PTS or improve QOL at 1 year (Odds Ratio (OR) 0.75; 95% CI, 0.38–1.50; *p* = 0.42) [89]. In the ultrasound-assisted CDT group, the median duration of symptoms prior to the procedure was 10 days [89]. The results reported according to the International Society of Thrombosis and Haemostasis (ISTH)—endorsed Villalta scoring method were remarkably similar to those of the iliofemoral DVT subgroup in ATTRACT [87,89].

In the above studies, there were no fatal or intracranial bleeds, but endovascular therapy did result in additional major bleeding events (1.4–5.6%). In ATTRACT, patients ≥65 years old experienced even-worse PTS prevention efficacy and most of the major bleeds.

Hence, the routine, relatively “unselected”, first-line use of endovascular DVT thrombolysis is not recommended [15,20,42,83,84,86,87,88,89,90]. However, it remains likely that patients with iliofemoral DVT derive improved symptom recovery and reduced PTS severity that translate into improved QOL with use of PCDT [82,87]. Additional work to identify discrete subgroups for whom routine first-line endovascular clot lysis is appropriate would be worthwhile [91]. At present, we suggest that PCDT may be reasonably considered in patients with acute iliofemoral DVT, severe symptoms, and low risk of bleeding (generally, patients less than 65 years of age), with careful discussion of bleeding risks and the possible benefits and due respect for patient preferences.

## 7. Management of Established PTS

PTS symptoms vary in degree and severity among patients but typically include some form of limb pain, aching, swelling, heaviness, tingling, and/or cramping with more concerning complications including the development of venous leg ulcers and dermatolipofasciosclerosis [20,92,93,94]. A careful approach to diagnosis in the clinic should be undertaken; it is not uncommon for a venous disease specialist to see patients previously misdiagnosed with neuropathic, arterial, or other causes of non-specific limb pain or swelling. Whether or not venous hypertension is present, general medical conditions (e.g., congestive heart failure, renal/liver disease, sleep apnea, osteoarthritis) and lymphedema can be superimposed. PTS should be recognized as a serious adverse outcome after DVT. In addition to considerably impairing a patient’s quality of life, PTS is also a financially costly condition for both the patient and the healthcare system [35]. In a retrospective, comparative cohort study, MacDougall et al. [95] estimated the annualized median cost of patients with DVT who developed PTS to be $20,569 as compared to $15,843 in matched patient who did not develop PTS. Because PTS can the last the entirety of a patient’s life, these costs quickly add up. Patient outcomes are most likely to be optimized when a multifaceted approach is taken with a strong understanding of the varying presentations of PTS as well as procedural and non-procedural treatment options.

The provider should seek to understand the clinical severity of the disease, impact on the patient’s life, anatomical distribution of the residual thrombus, presence of valvular reflux, thrombosis history, ongoing risk factors for recurrent thrombosis, and tolerance of previous therapies [96]. A physical exam should be performed that includes inspection of bilateral extremities for the presence of open ulcers, skin changes, edema, arterial pulses, and presence of superficial varicosities, as well as evaluation of the lower body wall, pelvis, and perineum for signs of venous collateralization. Information from venous duplex ultrasound (in particular, residual thrombus, valvular reflux, and common femoral vein Doppler waveforms) should be supplemented with information gained from history and physical exam.

PTS therapy should initially optimize the use of non-invasive therapies. A trial of compression therapy will be useful for most patients, since reduction of edema can contribute to reducing limb heaviness and even pain. In general, patients without a venous ulcer may be started on ECSs. If ECSs are not sufficient to alleviate symptoms, additional devices can be employed—either wearable venous return-assist devices or stationary intermittent edema pumps. In patients with venous ulcers, compression is the mainstay of effective therapy, with inelastic multilayer compression methods preferred. For some patients, pharmacological agents (e.g., rutosides, hidrosmin, and defibrotides, horse chestnut seed extract derivatives) may alleviate some PTS symptoms [97,98]. The American College of Chest Physicians (ACCP) guidelines have assigned a grade 2B recommendation for the use of pentoxifylline in addition to professional wound care and compression for patients with venous leg ulcers [99]. Structured exercise therapy can also be considered but would benefit from further study to establish its effectiveness, and protocol standardization, in the PTS population [96]. All patients should be encouraged to avoid smoking and to maintain a normal body weight and heart-healthy habits.

If conservative therapies fail to provide satisfactory results and severe PTS symptoms continue to cause substantial life impact, then the physician can begin to consider more active management strategies. In particular, a careful clinical assessment for two potentially reversible contributors to venous hypertension (saphenous reflux and iliac vein obstruction) should be performed.

First, chronic iliac vein obstruction causes significant elevation in ambulatory venous pressure, which can be reversed by endovascular recanalization [100]. There should be suspicion for iliac vein obstruction if one of the following is present: (a) pain or swelling of the entire limb during the initial DVT episode or with current daily PTS symptoms, (b) dominance of venous claudication, (c) history of imaging-proving thrombosis of the ipsilateral common femoral vein or iliac vein, (d) incomplete compressibility of the common femoral vein on ultrasound, and/or (e) lack of common femoral vein Doppler waveform phasicity compared to the contralateral limb [96].

In a systematic review and meta-analysis of 37 studies and 2869 patients, Razavi et al. [101] reported a technical success rate of stent placement in patients with iliofemoral obstruction that exceeded 90%, with a 1–2% occurrence of peri-procedure major bleeding, PE, or death (combined). More than two-thirds of treated patients experienced substantial improvement in limb pain, swelling, and ulcer healing [101]. However, 1-year primary patencies of venous stents for PTS have been modest (70–80%) [101]. Accordingly, there remain significant uncertainties regarding the long-term outcomes of stented veins and their utility in the management of PTS [101]. The ongoing Chronic Venous Thrombosis: Relief with Adjunctive Catheter-Directed Therapy (C-TRACT) trial is a National Institutes of Health funded, phase-III, multicenter, randomized, open-label, assessor-blinded controlled clinical trial that seeks to assess the effect of iliac vein stenting on the severity of PTS compared to standard non-invasive PTS therapy alone. Until there is strong evidence to guide informed decision making, it is wise to ensure that implantation of these permanent devices is performed in only the most severely affected patients, with risk and uncertainties clearly conveyed during informed consent. Based on available experience, patients with good inflow to the common femoral vein are most likely to experience sustained venous patency and clinical improvement after stent placement.

Valvular incompetence of the great saphenous vein is often also an important contributor to PTS sequelae following thrombosis and is reversible using catheter-based endovenous ablation techniques [96,100]. Two retrospective studies reported that the combination of iliofemoral venous stenting and great saphenous vein stripping or ablation was associated with a substantial improvement in limb swelling, pain, ulcer healing, and/or quality of life [96,102]. There is ongoing interest in developing a percutaneous solution (e.g., prosthetic valve) to the problem of deep venous valvular reflux, but no device has yet been shown to be efficacious and safe.

If all else fails and a patient continues to exhibit unremitting symptoms associated with severe PTS, then surgical consultation may be considered in select cases. For chronic venous obstruction, contemporary case series suggest mediocre patencies with use of venous–venous bypass and arteriovenous fistula creation in highly experienced hands. Surgical attempts to alleviate deep venous valvular reflux have been poorly studied, with just a few case series describing single-center experiences with segmental vein valve transfers or venous transposition [98]. Overall, deep venous valvular reflux remains a stubborn problem that often exacerbates chronic PTS sequelae.

## 8. Conclusions and Future Directions

The management of acute DVT and chronic PTS remains complex, with many possible avenues of improvement. Most importantly, the pathophysiology of PTS remains poorly understood—it is hoped that future studies will include evaluation of genomic factors and other biomarkers to shed light on who is most likely to develop PTS, progress in severity, and benefit from interventions.

An important short-term goal is to continue to educate the medical community on the importance of PTS to patients. It is now many years since PTS was recognized as a major contributor to poor QOL in DVT patients, yet it continues to be neglected as a study outcome of high priority. Regulatory agencies continue to be willing to approve new drugs for DVT without requiring quality assessments of their impact upon PTS occurrence and severity. Clinical practice guidelines continue to determine the need for anticoagulant therapy based mainly on the balance between recurrent VTE and major bleeding, without consideration for the impact of PTS upon patients’ lives.

It is also manifestly clear that patients with iliofemoral DVT constitute a distinctive subgroup that experiences more recurrent VTE, more PTS, and more severe PTS. Increased education is needed to ensure that these patients are identified as “high-risk” at diagnosis, that they receive closer early monitoring to ensure effective anticoagulation and symptom control, and to consider treatment escalation when appropriate. Treatment studies should routinely report outcomes separately in patients with iliofemoral DVT, since they may differ from those of other patients with proximal DVT.

At present, outcome assessment for patients with DVT is performed in a cross-sectional manner, with use of validated measures applied just a few times during patient follow-up. However, PTS is an ongoing malady that affects patients every day. Technology has progressed to where it may be possible to develop more innovative ways to capture the overall disease burden via patient-reported outcome reporting or data capture via sensors and like methods. After appropriate validation studies, such tools could be very useful to providers and researchers.

Regarding the use of currently available endovascular treatment options, randomized trials have clarified that selective use, rather than routine use, is most appropriate. Patients with highly symptomatic involvement of the iliofemoral, axillosubclavian, and caval veins should be the focus. Additional analyses would be helpful to determine whether specific subgroups of patients with iliofemoral DVT are most amenable to benefit and could create a case for routine, first-line use. Until then, therapy should be delivered on a highly individualized basis, with a focus on ensuring treatment safety, using best practices to optimize efficacy, and ensuring a patient voice in decision-making.

## References

[1] Vedantham S., Grassi C.J., Ferral H., Patel N.H., Thorpe P.E., Antonacci V.P., Janne d′Othée B.M., Hofmann L.V., Cardella J.F., Kundu S. (2006). Reporting Standards for Endovascular Treatment of Lower Extremity Deep Vein Thrombosis. J. Vasc. Interv. Radiol..

[2] Thomas M., Hollingsworth A., Mofidi R. (2019). Endovascular Management of Acute Lower Limb Deep Vein Thrombosis: A Systematic Review and Meta-analysis. Ann. Vasc. Surg..

[3] Bendix S.D., Nolan R., Banipal S., Oppat W.F. (2019). Posterior tibial vein approach to catheter-directed thrombolysis for iliofemoral deep venous thrombosis. J. Vasc. Surg. Venous Lymphat. Disord..

[4] Farrell J.J., Sutter C., Tavri S., Patel I. (2016). Incidence and interventions for post-thrombotic syndrome. Cardiovasc. Diagn. Ther..

[5] Nathan A.S., Giri J. (2019). Reexamining the Open-Vein Hypothesis for Acute Deep Venous Thrombosis. Circulation.

[6] Tovey C., Wyatt S. (2003). Diagnosis, investigation, and management of deep vein thrombosis. BMJ.

[7] Hood D.B., Weaver F.A., Modrall J.G., Yellin A.E. (1993). Advances in the treatment of phlegmasia cerulea dolens. Am. J. Surg..

[8] Ramirez P.J., Gohh R.Y., Kestin A., Monaco A.P., Morrissey P.E. (2002). Renal allograft loss due to proximal extension of ileofemoral deep venous thrombosis. Clin. Transplant..

[9] Wang R., Meng Q., Qu L., Wu X., Sun N., Jin X. (2013). Treatment of Budd-Chiari syndrome with inferior vena cava thrombosis. Exp. Ther. Med..

[10] Kim Y.J. (2019). Red flag rules for knee and lower leg differential diagnosis. Ann. Transl. Med..

[11] Rabinovich A., Kahn S.R. (2014). How to predict and diagnose postthrombotic syndrome. Pol. Arch. Intern. Med..

[12] Ende-Verhaar Y.M., Tick L.W., Klok F.A., Huisman M.V., Rosendaal F.R., le Cessie S., Cannegieter S.C. (2019). Post-thrombotic syndrome: Short and long-term incidence and risk factors. Thromb. Res..

[13] Schleimer K., Barbati M.E., Grommes J., Hoeft K., Toonder I.M., Wittens C.H.A., Jalaie H. (2019). Update on diagnosis and treatment strategies in patients with post-thrombotic syndrome due to chronic venous obstruction and role of endovenous recanalization. J. Vasc. Surg. Venous Lymphat. Disord..

[14] Vedantham S. (2008). Interventions for Deep Vein Thrombosis: Reemergence of a Promising Therapy. Am. J. Med..

[15] Kearon C., Akl E.A., Ornelas J., Blaivas A., Jimenez D., Bounameaux H., Huisman M., King C.S., Morris T.A., Sood N. (2016). Antithrombotic Therapy for VTE Disease: CHEST Guideline and Expert Panel Report. Chest.

[16] Kearon C., Akl E.A., Comerota A.J., Prandoni P., Bounameaux H., Goldhaber S.Z., Nelson M.E., Wells P.S., Gould M.K., Dentali F. (2012). Antithrombotic Therapy for VTE Disease: Antithrombotic Therapy and Prevention of Thrombosis, 9th ed: American College of Chest Physicians Evidence-Based Clinical Practice Guidelines. Chest.

[17] Semba C.P., Dake M.D. (1994). Iliofemoral deep venous thrombosis: Aggressive therapy with catheter-directed thrombolysis. Radiology.

[18] Endig H., Michalski F., Beyer-Westendorf J. (2016). Deep Vein Thrombosis–Current Management Strategies. Clin. Med. Insights Ther..

[19] Jenkins J.S., Michael P. (2014). Deep Venous Thrombosis: An Interventionalist’s Approach. Ochsner J..

[20] Vedantham S., Goldhaber S.Z., Julian J.A., Kahn S.R., Jaff M.R., Cohen D.J., Magnuson E., Razavi M.K., Comerota A.J., Gornik H.L. (2017). Pharmacomechanical Catheter-Directed Thrombolysis for Deep-Vein Thrombosis. N. Engl. J. Med..

[21] Robert-Ebadi H., Righini M. (2017). Should we diagnose and treat distal deep vein thrombosis?. Hematol. Am. Soc. Hematol. Educ. Program.

[22] Douketis J.D., Crowther M.A., Foster G.A., Ginsberg J.S. (2001). Does the location of thrombosis determine the risk of disease recurrence in patients with proximal deep vein thrombosis?. Am. J. Med..

[23] Kahn S.R., Shbaklo H., Lamping D.L., Holcroft C.A., Shrier I., Miron M.J., Roussin A., Desmarais S., Joyal F., Kassis J. (2008). Determinants of health-related quality of life during the 2 years following deep vein thrombosis. J. Thromb. Haemost..

[24] Ginsberg J.S., Hirsh J., Julian J., Vander LaandeVries M., Magier D., MacKinnon B., Gent M. (2001). Prevention and Treatment of Postphlebitic Syndrome: Results of a 3-Part Study. Arch. Intern. Med..

[25] Kahn S.R., Hirsch A., Shrier I. (2002). Effect of Postthrombotic Syndrome on Health-Related Quality of Life After Deep Venous Thrombosis. Arch. Intern. Med..

[26] Kahn S.R., Ginsberg J.S. (2004). Relationship Between Deep Venous Thrombosis and the Postthrombotic Syndrome. Arch. Intern. Med..

[27] Prandoni P., Lensing A.W., Cogo A., Cuppini S., Villalta S., Carta M., Cattelan A.M., Polistena P., Bernardi E., Prins M.H. (1996). The long-term clinical course of acute deep venous thrombosis. Ann. Intern. Med..

[28] Van Dongen C.J.J., Prandoni P., Frulla M., Marchiori A., Prins M.H., Hutten B.A. (2005). Relation between quality of anticoagulant treatment and the development of the postthrombotic syndrome. J. Thromb. Haemost..

[29] Chitsike R.S., Rodger M.A., Kovacs M.J., Betancourt M.T., Wells P.S., Anderson D.R., Chagnon I., G L.E.G., Solymoss S., Crowther M.A. (2012). Risk of post-thrombotic syndrome after subtherapeutic warfarin anticoagulation for a first unprovoked deep vein thrombosis: Results from the REVERSE study. J. Thromb. Haemost..

[30] Kahn S.R. (2016). The post-thrombotic syndrome. Hematology.

[31] Kahn S.R., Shapiro S., Wells P.S., Rodger M.A., Kovacs M.J., Anderson D.R., Tagalakis V., Houweling A.H., Ducruet T., Holcroft C. (2014). Compression stockings to prevent post-thrombotic syndrome: A randomised placebo-controlled trial. Lancet.

[32] Prandoni P., Lensing A.W., Prins M.H., Frulla M., Marchiori A., Bernardi E., Tormene D., Mosena L., Pagnan A., Girolami A. (2004). Below-knee elastic compression stockings to prevent the post-thrombotic syndrome: A randomized, controlled trial. Ann. Intern. Med..

[33] Ferreira T., Huber S.C., de Moraes Martinelli B., Junior A.L., Menezes F.H., Orsi F.A., Bittar L.F., de Oliveira L.F.G., Sodre L.R., Mello T.T. (2019). Low prevalence of Post-thrombotic syndrome in patients treated with rivaroxaban. Vasc. Pharmacol..

[34] Cheung Y.W., Middeldorp S., Prins M.H., Pap A.F., Lensing A.W., Ten Cate-Hoek A.J., Villalta S., Milan M., Beyer-Westendorf J., Verhamme P. (2016). Post-thrombotic syndrome in patients treated with rivaroxaban or enoxaparin/vitamin K antagonists for acute deep-vein thrombosis. A post-hoc analysis. Thromb. Haemost..

[35] Pikovsky O., Rabinovich A. (2018). Prevention and treatment of the post-thrombotic syndrome. Thromb. Res..

[36] Søgaard M., Nielsen P.B., Skjøth F., Kjældgaard J.N., Coleman C.I., Larsen T.B. (2018). Rivaroxaban Versus Warfarin and Risk of Post-Thrombotic Syndrome Among Patients with Venous Thromboembolism. Am. J. Med..

[37] Lozano F., Trujillo-Santos J., Barrón M., Gallego P., Babalis D., Santos M., Falgá C., Monreal M. (2014). Home versus in-hospital treatment of outpatients with acute deep venous thrombosis of the lower limbs. J. Vasc. Surg..

[38] Condliffe R. (2016). Pathways for outpatient management of venous thromboembolism in a UK centre. Thromb. J..

[39] Mol G.C., van de Ree M.A., Klok F.A., Tegelberg M.J.A.M., Sanders F.B.M., Koppen S., de Weerdt O., Koster T., Hovens M.M.C., Kaasjager H.A.H. (2016). One versus two years of elastic compression stockings for prevention of post-thrombotic syndrome (OCTAVIA study): Randomised controlled trial. BMJ.

[40] ten Cate-Hoek A.J., Amin E.E., Bouman A.C., Meijer K., Tick L.W., Middeldorp S., Mostard G.J.M., ten Wolde M., van den Heiligenberg S.M., van Wissen S. (2018). Individualised versus standard duration of elastic compression therapy for prevention of post-thrombotic syndrome (IDEAL DVT): A multicentre, randomised, single-blind, allocation-concealed, non-inferiority trial. Lancet Haematol..

[41] Musil D. (2018). Outpatient treatment of proximal deep vein thrombosis. Vnitr. Lek..

[42] Vedantham S. (2017). Thrombectomy and thrombolysis for the prevention and treatment of postthrombotic syndrome. Hematol. Am. Soc. Hematol. Educ. Program.

[43] Liu Z., Tao X., Chen Y., Fan Z., Li Y. (2015). Bed Rest versus Early Ambulation with Standard Anticoagulation in The Management of Deep Vein Thrombosis: A Meta-Analysis. PLoS ONE.

[44] Partsch H., Kaulich M., Mayer W. (2004). Immediate mobilisation in acute vein thrombosis reduces post-thrombotic syndrome. Int. Angiol..

[45] Hansrani V., Khanbhai M., McCollum C., Islam M.S. (2017). The Diagnosis and Management of Early Deep Vein Thrombosis. Thrombosis and Embolism: From Research to Clinical Practice: Volume 1.

[46] Oklu R., Wicky S. (2013). Catheter-directed thrombolysis of deep venous thrombosis. Semin. Thromb. Hemost..

[47] Behravesh S., Hoang P., Nanda A., Wallace A., Sheth R.A., Deipolyi A.R., Memic A., Naidu S., Oklu R. (2017). Pathogenesis of Thromboembolism and Endovascular Management. Thrombosis.

[48] Watson L.I., Armon M.P. (2004). Thrombolysis for acute deep vein thrombosis. Cochrane Database Syst. Rev..

[49] Plate G., Akesson H., Einarsson E., Ohlin P., Eklof B. (1990). Long-term results of venous thrombectomy combined with a temporary arterio-venous fistula. Eur. J. Vasc. Surg..

[50] Prandoni P., Lensing A.W., Prins M.H., Bernardi E., Marchiori A., Bagatella P., Frulla M., Mosena L., Tormene D., Piccioli A. (2002). Residual venous thrombosis as a predictive factor of recurrent venous thromboembolism. Ann. Intern. Med..

[51] Young L., Ockelford P., Milne D., Rolfe-Vyson V., Mckelvie S., Harper P. (2006). Post-treatment residual thrombus increases the risk of recurrent deep vein thrombosis and mortality. J. Thromb. Haemost..

[52] Hull R.D., Marder V.J., Mah A.F., Biel R.K., Brant R.F. (2005). Quantitative assessment of thrombus burden predicts the outcome of treatment for venous thrombosis: A systematic review. Am. J. Med..

[53] Prandoni P., Frulla M., Sartor D., Concolato A., Girolami A. (2005). Vein abnormalities and the post-thrombotic syndrome. J. Thromb. Haemost..

[54] Goldhaber S.Z., Buring J.E., Lipnick R.J., Hennekens C.H. (1984). Pooled analyses of randomized trials of streptokinase and heparin in phlebographically documented acute deep venous thrombosis. Am. J. Med..

[55] Arnesen H., Høiseth A., Ly B. (1982). Streptokinase or Heparin in the Treatment of Deep Vein Thrombosis. Acta Med. Scand..

[56] Okrent D., Messersmith R., Buckman J. (1991). Transcatheter fibrinolytic therapy and angioplasty for left iliofemoral venous thrombosis. J. Vasc. Interv. Radiol..

[57] Vedantham S., Sista A.K., Klein S.J., Nayak L., Razavi M.K., Kalva S.P., Saad W.E., Dariushnia S.R., Caplin D.M., Chao C.P. (2014). Quality Improvement Guidelines for the Treatment of Lower-Extremity Deep Vein Thrombosis with Use of Endovascular Thrombus Removal. J. Vasc. Interv. Radiol..

[58] Sharifi M., Bay R., Nowroozi S., Bentz S., Valeros G., Memari S. (2013). Catheter-directed Thrombolysis with Argatroban and tPA for Massive Iliac and Femoropopliteal Vein Thrombosis. Cardiovasc. Interv. Radiol..

[59] Badreldin H.A., Rimsans J., Connors J.M., Wiviott S.D. (2018). Use of systemic bivalirudin with catheter-directed thrombolysis in a patient with heparin-induced thrombocytopenia: A case report. Catheter. Cardiovasc. Interv..

[60] AS E.L., AlQattan A.S., Elashaal E., AlSadery H., AlGhanmi I., Aldhafery B.F. (2019). The ugly face of deep vein thrombosis: Phlegmasia Cerulea Dolens-Case report. Int. J. Surg. Case Rep..

[61] Oguzkurt L., Ozkan U., Demirturk O.S., Gur S. (2011). Endovascular Treatment of Phlegmasia Cerulea Dolens with Impending Venous Gangrene: Manual Aspiration Thrombectomy as the First-Line Thrombus Removal Method. Cardiovasc. Interv. Radiol..

[62] Chaochankit W., Akaraborworn O. (2018). Phlegmasia Cerulea Dolens with Compartment Syndrome. Ann. Vasc. Dis..

[63] Yang S.-S., Yun W.-S. (2016). Surgical Thrombectomy for Phlegmasia Cerulea Dolens. Vasc. Spec. Int..

[64] Tung C.S., Soliman P.T., Wallace M.J., Wolf J.K., Bodurka D.C. (2007). Successful catheter-directed venous thrombolysis in phlegmasia cerulea dolens. Gynecol. Oncol..

[65] Lessne M.L., Bajwa J., Hong K. (2012). Fatal Reperfusion Injury after Thrombolysis for Phlegmasia Cerulea Dolens. J. Vasc. Interv. Radiol..

[66] Kim H.S., Fine D.M., Atta M.G. (2006). Catheter-directed Thrombectomy and Thrombolysis for Acute Renal Vein Thrombosis. J. Vasc. Interv. Radiol..

[67] Jaar B.G., Kim H.S., Samaniego M.D., Lund G.B., Atta M.G. (2002). Percutaneous mechanical thrombectomy: A new approach in the treatment of acute renal-vein thrombosis. Nephrol. Dial. Transplant..

[68] Melamed M.L., Kim H.S., Jaar B.G., Molmenti E., Atta M.G., Samaniego M.D. (2005). Combined Percutaneous Mechanical and Chemical Thrombectomy for Renal Vein Thrombosis in Kidney Transplant Recipients. Am. J. Transplant..

[69] Ding P.X., He X., Han X.W., Zhang Y., Wu Y., Liang X.X., Liu C. (2018). An Individualised Strategy and Long-Term Outcomes of Endovascular Treatment of Budd–Chiari Syndrome Complicated by Inferior Vena Cava Thrombosis. Eur. J. Vasc. Endovasc. Surg..

[70] Kuo G.P., Brodsky R.A., Kim H.S. (2006). Catheter-directed thrombolysis and thrombectomy for the Budd-Chiari syndrome in paroxysmal nocturnal hemoglobinuria in three patients. J. Vasc. Interv. Radiol..

[71] Mitsuoka H., Saito T., Higashi S. (2014). Stepwise angioplasty and catheter directed thrombolysis for budd-Chiari syndrome complicated with floating thrombus in inferior vena cava. Ann. Vasc. Dis..

[72] Zimmerman S., Davis M. (2018). Rapid Fire: Superior Vena Cava Syndrome. Emerg. Med. Clin. N. Am..

[73] Rachapalli V., Boucher L.-M. (2014). Superior Vena Cava Syndrome: Role of the Interventionalist. Can. Assoc. Radiol. J..

[74] Shatila W., Almanfi A., Massumi M., Dougherty K.G., Parekh D.R., Strickman N.E. (2016). Endovascular Treatment of Superior Vena Cava Syndrome via Balloon-in-Balloon Catheter Technique with a Palmaz Stent. Tex. Heart Inst. J..

[75] Sfyroeras G.S., Antonopoulos C.N., Mantas G., Moulakakis K.G., Kakisis J.D., Brountzos E., Lattimer C.R., Geroulakos G. (2017). A Review of Open and Endovascular Treatment of Superior Vena Cava Syndrome of Benign Aetiology. Eur. J. Vasc. Endovasc. Surg..

[76] Kee S.T., Kinoshita L., Razavi M.K., Nyman U.R., Semba C.P., Dake M.D. (1998). Superior vena cava syndrome: Treatment with catheter-directed thrombolysis and endovascular stent placement. Radiology.

[77] Nunnelee J.D. (2007). Superior vena cava syndrome. J. Vasc. Nurs..

[78] Flounders J.A. (2003). Oncology emergency modules: Superior vena cava syndrome. Oncol. Nurs. Forum.

[79] Ghanavati R., Amiri A., Ansarinejad N., Hajsadeghi S., Riahi Beni H., Sezavar S.H. (2017). Successful Treatment of a Catheter-Induced Superior Vena Cava Syndrome through Catheter-Directed Thrombolysis: A Case Report. J. Tehran Heart Cent..

[80] Hangge P., Rotellini-Coltvet L., Deipolyi A.R., Albadawi H., Oklu R. (2017). Paget-Schroetter syndrome: Treatment of venous thrombosis and outcomes. Cardiovasc. Diagn. Ther..

[81] Taylor J.M., Telford R.J., Kinsella D.C., Watkinson A.F., Thompson J.F. (2013). Long-term clinical and functional outcome following treatment for Paget–Schroetter syndrome. Br. J. Surg..

[82] Kahn S.R., Julian J.A., Kearon C., Gu C.-S., Cohen D.J., Magnuson E.A., Comerota A.J., Goldhaber S.Z., Jaff M.R., Razavi M.K. (2020). Quality of life after pharmacomechanical catheter-directed thrombolysis for proximal deep venous thrombosis. J. Vasc. Surg. Venous Lymphat. Disord..

[83] Enden T., Haig Y., Klow N.E., Slagsvold C.E., Sandvik L., Ghanima W., Hafsahl G., Holme P.A., Holmen L.O., Njaastad A.M. (2012). Long-term outcome after additional catheter-directed thrombolysis versus standard treatment for acute iliofemoral deep vein thrombosis (the CaVenT study): A randomised controlled trial. Lancet.

[84] Haig Y., Enden T., Grøtta O., Kløw N.-E., Slagsvold C.-E., Ghanima W., Sandvik L., Hafsahl G., Holme P.A., Holmen L.O. (2016). Post-thrombotic syndrome after catheter-directed thrombolysis for deep vein thrombosis (CaVenT): 5-year follow-up results of an open-label, randomised controlled trial. Lancet Haematol..

[85] Enden T., Resch S., White C., Wik H.S., Kløw N.E., Sandset P.M. (2013). Cost-effectiveness of additional catheter-directed thrombolysis for deep vein thrombosis. J. Thromb. Haemost. JTH.

[86] Kearon C., Gu C.-S., Julian J.A., Goldhaber S.Z., Comerota A.J., Gornik H.L., Murphy T.P., Lewis L., Kahn S.R., Kindzelski A.L. (2019). Pharmacomechanical Catheter-Directed Thrombolysis in Acute Femoral–Popliteal Deep Vein Thrombosis: Analysis from a Stratified Randomized Trial. Thromb. Haemost..

[87] Comerota A.J., Kearon C., Gu C.S., Julian J.A., Goldhaber S.Z., Kahn S.R., Jaff M.R., Razavi M.K., Kindzelski A.L., Bashir R. (2019). Endovascular Thrombus Removal for Acute Iliofemoral Deep Vein Thrombosis. Circulation.

[88] Magnuson E.A., Chinnakondepalli K., Vilain K., Kearon C., Julian J.A., Kahn S.R., Goldhaber S.Z., Jaff M.R., Kindzelski A.L., Herman K. (2019). Cost-Effectiveness of Pharmacomechanical Catheter-Directed Thrombolysis Versus Standard Anticoagulation in Patients With Proximal Deep Vein Thrombosis. Circ. Cardiovasc. Qual. Outcomes.

[89] Notten P., ten Cate-Hoek A.J., Arnoldussen C.W.K.P., Strijkers R.H.W., de Smet A.A.E.A., Tick L.W., van de Poel M.H.W., Wikkeling O.R.M., Vleming L.-J., Koster A. (2020). Ultrasound-accelerated catheter-directed thrombolysis versus anticoagulation for the prevention of post-thrombotic syndrome (CAVA): A single-blind, multicentre, randomised trial. Lancet Haematol..

[90] Enden T., Wik H.S., Kvam A.K., Haig Y., Kløw N.E., Sandset P.M. (2013). Health-related quality of life after catheter-directed thrombolysis for deep vein thrombosis: Secondary outcomes of the randomised, non-blinded, parallel-group CaVenT study. BMJ Open.

[91] Vedantham S. (2020). Using it wisely: Catheter-directed thrombolysis for deep vein thrombosis. Lancet Haematol..

[92] Kahn S.R. (2009). How I treat postthrombotic syndrome. Blood.

[93] Vazquez S.R., Kahn S.R. (2010). Postthrombotic Syndrome. Circulation.

[94] Schleimer K., Barbati M.E., Gombert A., Wienert V., Grommes J., Jalaie H. (2016). The Treatment of Post-Thrombotic Syndrome. Dtsch. Ärztebl. Int..

[95] MacDougall D.A., Feliu A.L., Boccuzzi S.J., Lin J. (2006). Economic burden of deep-vein thrombosis, pulmonary embolism, and post-thrombotic syndrome. Am. J. Health Syst. Pharm..

[96] Nayak L., Vedantham S. (2012). Multifaceted management of the postthrombotic syndrome. Semin. Interv. Radiol..

[97] Pittler M.H., Ernst E. (2012). Horse chestnut seed extract for chronic venous insufficiency. Cochrane Database Syst. Rev..

[98] Kahn S.R., Comerota A.J., Cushman M. (2014). The postthrombotic syndrome: Evidence-based prevention, diagnosis, and treatment strategies: A scientific statement from the American Heart Association. Circulation.

[99] Kearon C., Kahn S.R., Agnelli G., Goldhaber S., Raskob G.E., Comerota A.J. (2008). Antithrombotic therapy for venous thromboembolic disease: American College of Chest Physicians Evidence-Based Clinical Practice Guidelines (8th Edition). Chest.

[100] Vedantham S. (2009). Valvular dysfunction and venous obstruction in the post-thrombotic syndrome. Thromb. Res..

[101] Razavi M.K., Jaff M.R., Miller L.E. (2015). Safety and Effectiveness of Stent Placement for Iliofemoral Venous Outflow Obstruction: Systematic Review and Meta-Analysis. Circ. Cardiovasc. Interv..

[102] Neglen P., Hollis K.C., Raju S. (2006). Combined saphenous ablation and iliac stent placement for complex severe chronic venous disease. J. Vasc. Surg..

